# Somatic Pairing of Chromosome 19 in Renal Oncocytoma Is Associated with Deregulated ELGN2-Mediated Oxygen-Sensing Response

**DOI:** 10.1371/journal.pgen.1000176

**Published:** 2008-09-05

**Authors:** Julie M. Koeman, Ryan C. Russell, Min-Han Tan, David Petillo, Michael Westphal, Katherine Koelzer, Julie L. Metcalf, Zhongfa Zhang, Daisuke Matsuda, Karl J. Dykema, Heather L. Houseman, Eric J. Kort, Laura L. Furge, Richard J. Kahnoski, Stéphane Richard, Annick Vieillefond, Pamela J. Swiatek, Bin Tean Teh, Michael Ohh, Kyle A. Furge

**Affiliations:** 1 Laboratory of Germline Modification and Cytogenetics, Van Andel Research Institute, Grand Rapids, Michigan, United States of America; 2 Department of Laboratory Medicine and Pathobiology, University of Toronto, Toronto, Ontario, Canada; 3 Laboratory of Cancer Genetics, Van Andel Research Institute, Grand Rapids, Michigan, United States of America; 4 NCCS-VARI Translational Research Laboratory, National Cancer Centre of Singapore, Singapore, Singapore; 5 Department of Medical Oncology, National Cancer Center of Singapore, Singapore, Singapore; 6 Centre for Molecular Epidemiology, Department of Community, Occupational and Family Medicine, National University of Singapore, Singapore, Singapore; 7 Laboratory of Computational Biology, Van Andel Research Institute, Grand Rapids, Michigan, United States of America; 8 Laboratory of Molecular Epidemiology, Van Andel Research Institute, Grand Rapids, Michigan, United States of America; 9 Department of Urology, Spectrum Health Hospital, Grand Rapids, Michigan, United States of America; 10 Génétique Oncologique EPHE, French Kidney Cancer Consortium, AP-HP, Service d'Urologie, Le Kremlin-Bicêtre, France; 11 CNRS FRE 2939, Institut Gustave Roussy, Villejuif, France; 12 French Kidney Cancer Consortium, Paris, France; 13 Laboratoire d'Anatomie Pathologique, AP-HP, Hôpital Cochin, Paris, France; Fred Hutchinson Cancer Research Center, United States of America

## Abstract

Chromosomal abnormalities, such as structural and numerical abnormalities, are a common occurrence in cancer. The close association of homologous chromosomes during interphase, a phenomenon termed somatic chromosome pairing, has been observed in cancerous cells, but the functional consequences of somatic pairing have not been established. Gene expression profiling studies revealed that somatic pairing of chromosome 19 is a recurrent chromosomal abnormality in renal oncocytoma, a neoplasia of the adult kidney. Somatic pairing was associated with significant disruption of gene expression within the paired regions and resulted in the deregulation of the prolyl-hydroxylase ELGN2, a key protein that regulates the oxygen-dependent degradation of hypoxia-inducible factor (HIF). Overexpression of ELGN2 in renal oncocytoma increased ubiquitin-mediated destruction of HIF and concomitantly suppressed the expression of several HIF-target genes, including the pro-death *BNIP3L* gene. The transcriptional changes that are associated with somatic pairing of chromosome 19 mimic the transcriptional changes that occur following DNA amplification. Therefore, in addition to numerical and structural chromosomal abnormalities, alterations in chromosomal spatial dynamics should be considered as genomic events that are associated with tumorigenesis. The identification of *EGLN2* as a significantly deregulated gene that maps within the paired chromosome region directly implicates defects in the oxygen-sensing network to the biology of renal oncocytoma.

## Author Summary

Together, renal oncocytoma and chromophobe renal cell carcinoma (RCC) account for approximately 10% of masses that are resected from the kidney. However, the molecular defects that are associated with the development of these neoplasias are not clear. Here, we take advantage of recent advances in genetics and computational analysis to screen for chromosomal abnormalities that are present in both renal oncocytoma and chromophobe RCC. We show that while chromophobe RCC cells contain an extra copy of chromosome 19, the renal oncoctyoma cells contain a rarely reported chromosomal abnormality. Both of these chromosomal abnormalities result in transcriptional disruptions of *EGLN2*, a gene that is located on chromosome 19 and is critical for the cellular response to changes in oxygen levels. Defects in oxygen sensing are found in other types of kidney tumors, and the identification of *EGLN2* directly implicates defects in the oxygen-sensing network in these neoplasias as well. These findings are important because the chromosomal defect present in renal oncocytomas may also be present in other tumor cells. In addition, deregulation of *EGLN2* reveals a unique way in which perturbations in oxygen-sensing are associated with disease.

## Introduction

Cellular adaptation to changes in oxygen tension is vital for the integrity, maintenance and survival of cells. Hypoxia-inducible factor (HIF), the major transcription factor of the ubiquitous oxygen-sensing pathway, is a heterodimer composed of α and β subunits [1]. While HIFβ is constitutively expressed and stable, HIFα is oxygen-labile by the virtue of the oxygen-dependent degradation (ODD) domain, which undergoes rapid oxygen-dependent ubiquitin-mediated destruction [2]–[5]. Thus, the stability of HIFα dictates the transcriptional activity of HIF [6]. Critical regulators of HIFα stability are the prolyl-hydroxylase domain-containing enzymes (PHD/EGLNs) that hydroxylate HIFα on conserved prolines within the ODD domain in the presence of oxygen [7],[8]. Hydroxylated HIFα is recognized by the von Hippel-Lindau (VHL) protein. VHL is the substrate-conferring component of an E3 ubiquitin ligase called ECV (Elongins/Cul2/VHL) that specifically polyubiquitinates prolyl-hydroxylated HIFα for subsequent destruction via the 26S proteasome.

Deregulation of HIFα regulatory proteins has been strongly associated with cancer development. Germline inheritance of a faulty *VHL* allele on chromosome 3p25 is the cause of VHL disease, characterized by a high frequency of clear cell renal cell carcinoma (RCC), cerebellar hemangioblastoma, pheochromocytoma, and retinal angioma [9]. Inactivation of the remaining wild-type VHL allele in a susceptible cell leads to tumor formation. Somatic biallelic inactivation of *VHL* is also responsible for the development of sporadic clear-cell RCCs, the predominant form of adult kidney cancer [10]–[12]. Cells that are devoid of functional VHL show elevated expression of numerous hypoxia-inducible genes due to a failure to degrade HIFα. In addition to VHL, deregulation of the PHD/EGLN family of prolyl-hydroxylases have also been associated with abnormal cell growth. Development of erythrocytosis, characterized by an excess of erythrocytes, has been associated with inactivating germline mutations in PHD2/EGLN1 [13],[14]. Pheochromocytoma, a neuroendocrine tumor of the medulla of the adrenal glands, is linked with deregulation of PHD3/EGLN3 [15].

While biallelic inactivation of VHL is found in the majority of clear cell RCCs, kidney cancer is a heterogeneous disease that can be divided into several subtypes based on morphological and cytogenetic features [16],[17]. Chromophobe RCC and renal oncocytoma are two related kidney tumors that together account for approximately 10% of all renal masses. In contrast to clear cell RCC, *VHL* mutations and/or increased expression of hypoxia-inducible genes are not found in these tumor subtypes and molecular genetic defects that are associated with tumor development remain unclear. Identification of molecular genetic defects in renal oncocytoma is particularly challenging as these cells are often described as karyotypically normal and the presence of cytogenetically abnormal regions in which to search for tumor modifying genes is rare in this tumor subtype.

To identify molecular defects associated with renal tumor development, we analyzed gene expression data from a variety of kidney tumors. This analysis revealed that renal oncocytoma and chromophobe RCC have a striking transcriptional disruption along chromosome 19. While in chromophobe RCC the disruption reflected a chromosome 19 amplification, in the renal oncocytoma cells the disruption reflected the close association, or pairing, of chromosome 19q in interphase. *EGLN2* located within the paired region was dramatically overexpressed in renal oncocytoma cells and was associated with the deregulation of numerous hypoxia-inducible genes including a pro-death *BNIP3L*. Thus, chromosome 19q pairing in renal oncocytoma unveils a unique mechanism of disrupting oxygen homeostasis via altering the expression of EGLN2.

## Results

**Figure 1:**
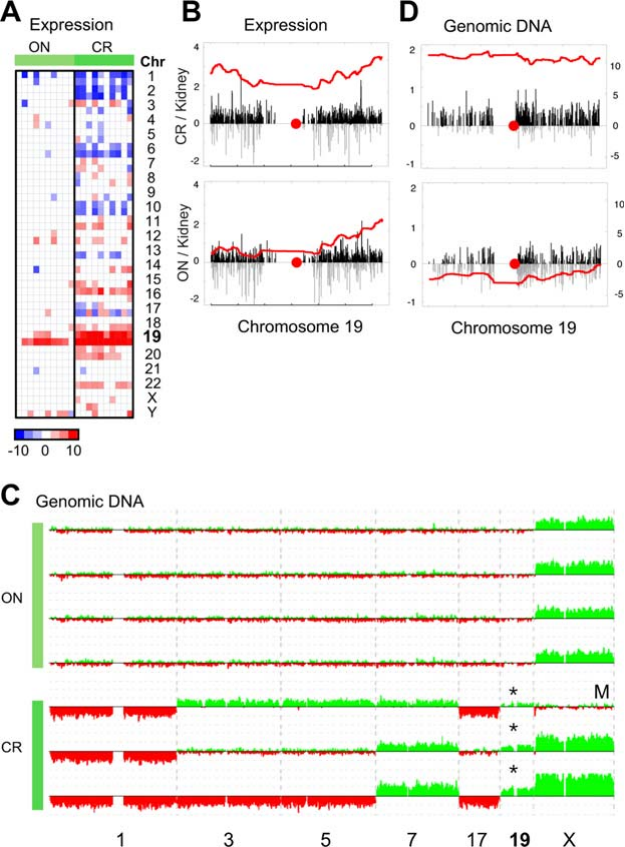
Transcriptional abnormalities in oncocytoma and chromophobe RCC. (A) Genomic regions that have significantly higher (red) or lower (blue) RNA production in renal oncocytoma (ON, *n* = 10) and chromophobe RCC (CR, *n* = 10) relative to non-diseased kidney (*n* = 10) were identified using the comparative genomic microarray analysis (CGMA) method as described in the Materials and Methods. Plotted is the resulting *t*-statistic obtained for each chromosome arm. Only the most significant results are shown (*P*<0.005). (B) For each gene on chr 19, the average log_2_-transformed expression ratio comparing oncocytoma or chromophobe RCC to non-diseased kidney is plotted relative to genomic location. The red circle indicates the location of the centromere. A smoothing curve was fit to the log_2_-transformed data to highlight regions that contain a disproportionate number of up-regulated genes. (C) Genomic regions that have overall increased (green) or decreased (red) DNA copy number in renal oncocytoma (ON) and chromophobe RCC (CR). SNP-derived DNA copy numbers were computed as described in the Material and Methods. All tumor samples were obtained from female patients except of the single male sample indicated (M). (D) For each SNP on chr 19, the average log_2_-transformed DNA copy number ratio comparing oncocytoma (*n* = 4) or chromophobe RCC (*n* = 3) to a pooled normal reference is plotted relative to genomic location as described in (C).

Gene expression profiling data derived from renal oncocytomas and chromophobe RCCs was scanned for regional increases or decreases in RNA production, which often indicate the presence of chromosomal amplifications or deletions [18]–[24]. Consistent with previous cytogenetic studies, the renal oncocytoma cells were largely devoid of transcriptional abnormalities that would reflect a DNA amplification or deletion. In contrast, losses of chromosomes 1, 2, 6, 10, and 17 are frequently found in chromophobe RCC. In our chromophobe RCC samples, these well-established chromosomal losses were strongly reflected in the gene expression profiling data (Figure 1A). In addition, a transcriptional abnormality involving genes mapping to chr 19 was frequently identified in both the renal oncocytomas and the chromophobe RCCs but not other subtypes of RCC (Figure 1A and Figure S1). In renal oncocytomas, the transcriptional abnormality primarily involved the q arm of chromosome 19, while in chromophobe RCC the abnormality involved the entire chromosome (Figure 1A,B).

Regional increases in overall RNA production often indicate the presence of an underlying DNA amplification. As gain of chromosome 19 has not been previously reported as a recurrent abnormality in either renal oncocytoma or chromophobe RCC, DNA copy number analysis was performed on a subset of these samples using high-density single nucleotide polymorphism (SNP) arrays. From the SNP data, an amplification of the entirety of chromosome 19 was detected in the chromophobe RCC samples (Figure 1C,D). This whole-chromosome amplification was confirmed by fluorescence in-situ hybridization (FISH) using locus-specific probes that mapped to the p and q arms of chromosome 19 (Table S1). In contrast, no change in DNA copy number was detected in the renal oncocytoma samples (Figure 1C,D). As a positive control for the DNA copy number analysis, only oncocytoma (ON) samples derived from female patients were examined, and a relative gain of the X chromosome was clearly detected in these samples (Figure 1C).

**Figure 2:**
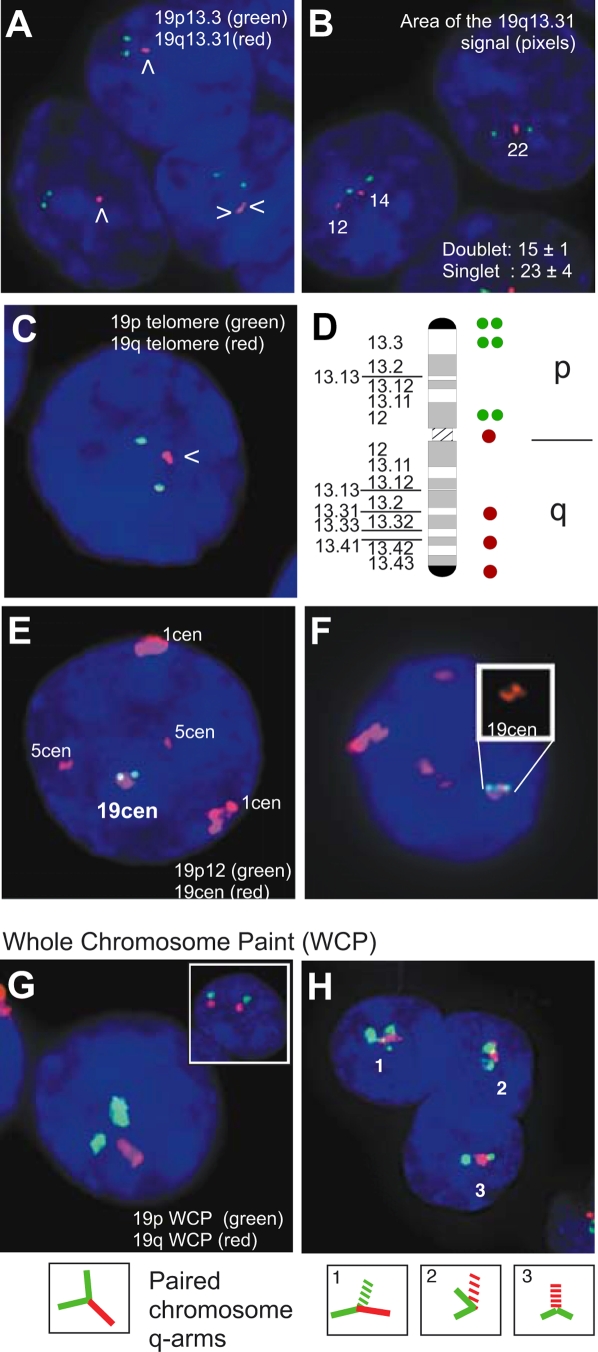
Somatic chromosome pairing in renal oncocytoma. Representative photomicrographs of tri-color interphase FISH on renal oncocytoma touch preparations. White arrows indicate large singlet or proximal doublet signals. In all images DAPI counterstaining is shown in blue. (A,B) Labeling with 19p13.3 (green) and 19q13.31 (red) probes. Image area of the 19q13.31 signal was quantified across multiple cells (*n* = 25) in the same image plane. Area mean and standard error are shown. (C,E,F) Labeling with 19p telomere (green) and 19q telomere (red) probes or 19p12 (green) and an alpha satellite probe for chr 19 that also cross-hybridizes to chromosomes 1 and 5 (red). Inset highlights centromeric pattern. (D) Schematic representation of frequently observed FISH patterns. (G,H) Whole-arm chromosome paint (WCP) for the chr 19 p-arm (red) and chr 19 q-arm (green). The inset shows a normal cell. Schematic representations of the paired chromosomes are shown below. Dashed lines represent chromosomal regions perpendicular to the plane of the image.

To determine the status of chromosome 19 in more detail in the renal oncocytoma cells, this chromosome was evaluated further using a panel of FISH probes. Two distinct and well-separated FISH signals, typical of diploid cells in interphase, were frequently observed when probes specific to the chr 19p arm were used (Figure 2 and Table S2). In contrast, a single, large FISH signal (singlet) or two FISH signals that were in close proximity (proximal doublet) were frequently observed when probes specific to the chr 19q arm were used. Approximately 35% of cells examined contained the singlet signal, while an additional 18% of cells contained proximal doublets (Table S2 and data not shown).

Semi-quantitative image analysis was used to examine the characteristics of the large FISH singlet (Figure 2B). This analysis demonstrated that the size of the singlet FISH signal was on average 1.5-fold larger than the size of two well-separated 19q FISH signals (*P* = 0.02). This large signal was observed using multiple probes directed against the q arm of the chromosome, including centromeric and telomeric probes (Figure 2C,E). The large FISH singlet had striking similarities to the FISH signals observed in studies of somatically paired chromosomes [25]–[27]. Somatic pairing refers to the close association of homologous chromosomes and is typically associated with chromosomes in meiotic prophase. However, somatic pairing has also been observed in interphase in normal human cells and some tumor cells [26], [28]–[32]. The presence of a large FISH singlet reflects the overlapping FISH signals generated from two chromosomal regions in very close proximity [26],[27]. The lack of evidence for a DNA copy number change coupled with the presence of large FISH singlets and proximal doublets using multiple locus-specific probes, suggested that chr 19q was somatically paired.

To confirm that the q arms of chr 19 were somatically paired in the renal oncocytoma cells, the p and q arms of chr 19 were visualized simultaneously using whole-arm chromosome painting (WCP). Using this approach, two distinct p arms, typical of diploid cells in interphase, were frequently observed in renal oncocytoma cells (Figure 2G,H and Table S2). However, the majority of cells contained a single q-arm signal that was located proximal to the two p-arm signals. While the diffuse nature of the WCP prevented the quantification the fluorescence signal, this pattern is consistent with the locus-specific FISH analysis and further indicates that the q arms of the chromosomes are in close proximity or are paired in these cells.

**Figure 3:**
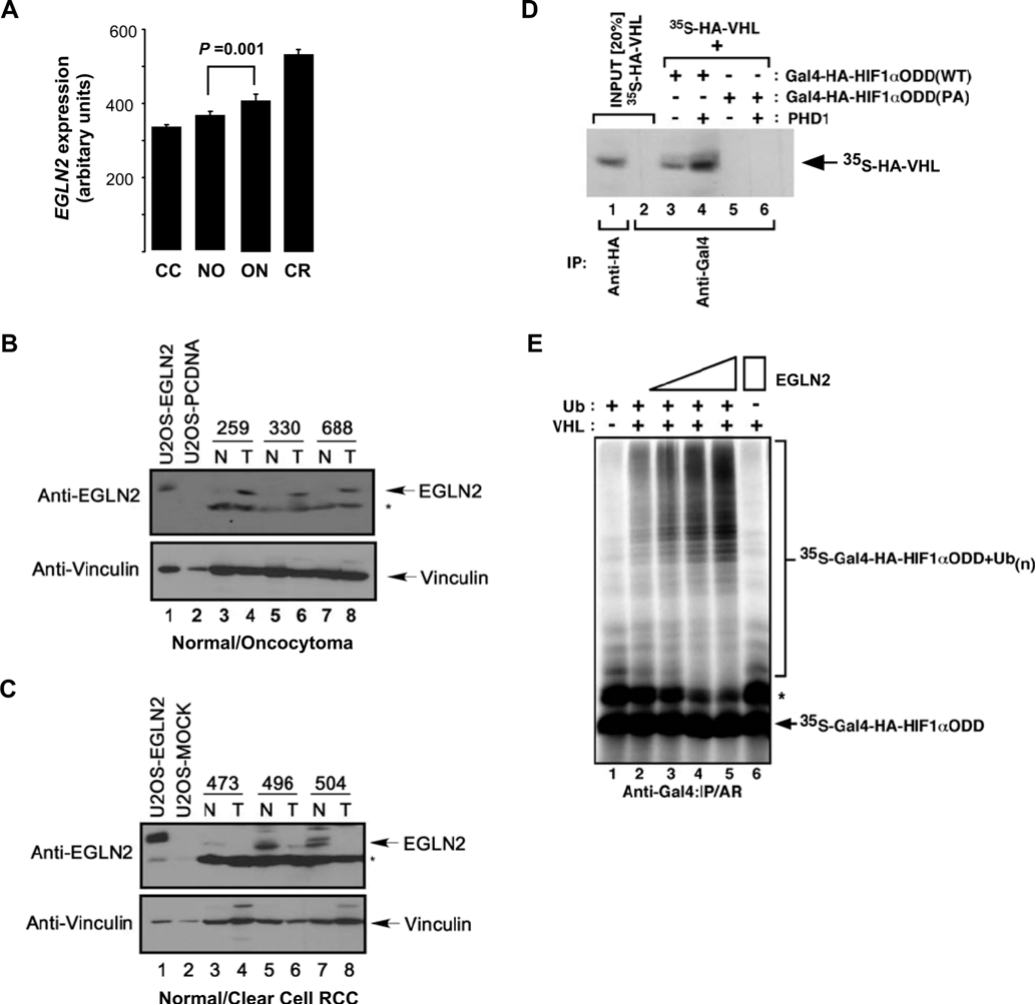
Overexpression of EGLN2 in renal oncocytoma. (A) Relative expression of EGLN2 as determined by gene expression microarray. (B) Anti-EGLN2 immunoblot analysis of whole-cell extracts prepared from oncocytoma (T) or patient-matched normal tissue (N) samples (lanes 3–8, top panel) and exogenously expressed EGLN2 controls (lanes 1–2, top panel). U2OS were transfected with plasmid containing human EGLN2 or empty vector alone (MOCK); EGLN2 appears as a single band of 45 kDa. Anti-vinculin immunoblot of whole-cell extracts was performed as a loading control. Asterisk (*) denotes background band. (C) Anti-EGLN2 immunoblot analysis of whole-cell extracts prepared from renal clear cell carcinoma (T) or patient-matched normal tissue (N) samples as described in (B) with the exception that a longer film exposure was required to reveal the EGLN2 protein in normal tissue. (D) In vitro translated Gal4-HA-HIF-1αODD(WT) and Gal4-HA-HIF-1αODD(PA) were treated with or without cellular extracts enriched for EGLN2 and mixed with in vitro translated ^35^S-labelled HA-VHL. Reaction mixtures were immunoprecipitated with anti-Gal4 antibody, bound proteins resolved on SDS-PAGE and visualized by autoradiography (lanes 3–6). In vitro translated ^35^S-labelled HA-VHL was also immunoprecipitated with anti-HA or anti-Gal4 antibody as input controls (lanes 1 and 2) and visualized by autoradiography. (E) In vitro ubiquitylation of ^35^S-labelled Gal4-HA-HIF-1αODD(WT) treated with increasing amounts of exogenous EGLN2 was performed in S100 cellular extracts devoid of VHL or reconstituted with VHL. All reaction mixtures except in lane 6 received purified ubiquitin. Reaction mixtures were immunoprecipitated with anti-Gal4 antibody, resolved on SDS-PAGE and visualized by autoradiography. * denotes uncharacterized modified Gal4-HA-HIF-1αODD; IP: immunoprecipitation; AR: autoradiography.

The changes in gene expression that accompanied the somatic pairing suggested that deregulation of a gene, or multiple genes, associated with tumor development mapped within the paired chr 19q region. As deregulation of the oxygen-sensing network is a common event in other types of sporadic renal cell carcinomas, genes associated with HIF regulation and that mapped to chr 19q were identified from the Entrez Gene database and tested for expression defects (see Materials and Methods). We also identified additional genes that were related to kidney-cancer via additional literature searching (Table S3). Both analyses identified EGLN2/PHD1 as a possible candidate gene in this region. To verify that the prolyl-hydroxylase EGLN2/PHD1 was significantly deregulated in renal oncocytoma cells, the level of EGLN2 protein was evaluated in these tumors (Figure 3A,B). Analysis of matched oncocytoma-normal tissue pairs revealed a dramatic increase in the level of EGLN2 in the oncocytoma tumors versus the level observed in corresponding normal tissue. Higher expression of EGLN2 was also observed in 2 of 3 chromophobe RCCs examined (Figure S2). These results are in contrast to the EGLN2 levels found in clear cell RCC. Consistent with the gene expression data, virtually no EGLN2 protein was detected in patient-derived clear cell RCC samples, while low basal amounts of EGLN2 were visualized by Western blot analysis in the matched normal samples (Figure 3 A,C).

EGLN2 is one of three prolyl-hydroxylases known to post-translationally modify HIFα, which is required for VHL-mediated destruction of HIFα. To address whether increased expression of EGLN2 influenced the binding and ubiquitination of HIF-1αODD via VHL, *in vitro* translated ^35^S-labeled HA-VHL and *in vitro* translated unlabeled Gal4-HA-HIF-1αODD were mixed in extracts in which EGLN2 was enriched (see Materials and Methods). Enrichment of EGLN2 led to an increased association of VHL to the wild-type ODD, but not to a mutated ODD in which a proline residue critical for VHL binding was changed to an alanine (P546A) (Figure 3D). In addition, an *in vitro* HIF-1αODD ubiquitination assay was performed to determine whether the increased VHL-HIF-1αODD association led to increased HIF-1αODD ubiquitination. Increased levels of EGLN2 resulted in a dose-dependent increase in VHL-mediated HIF-1αODD ubiquitination (Figure 3E). These results suggest that overexpression of EGLN2 in oncocytoma could further decrease the level of HIFα below the level observed in normal tissue.

**Figure 4:**
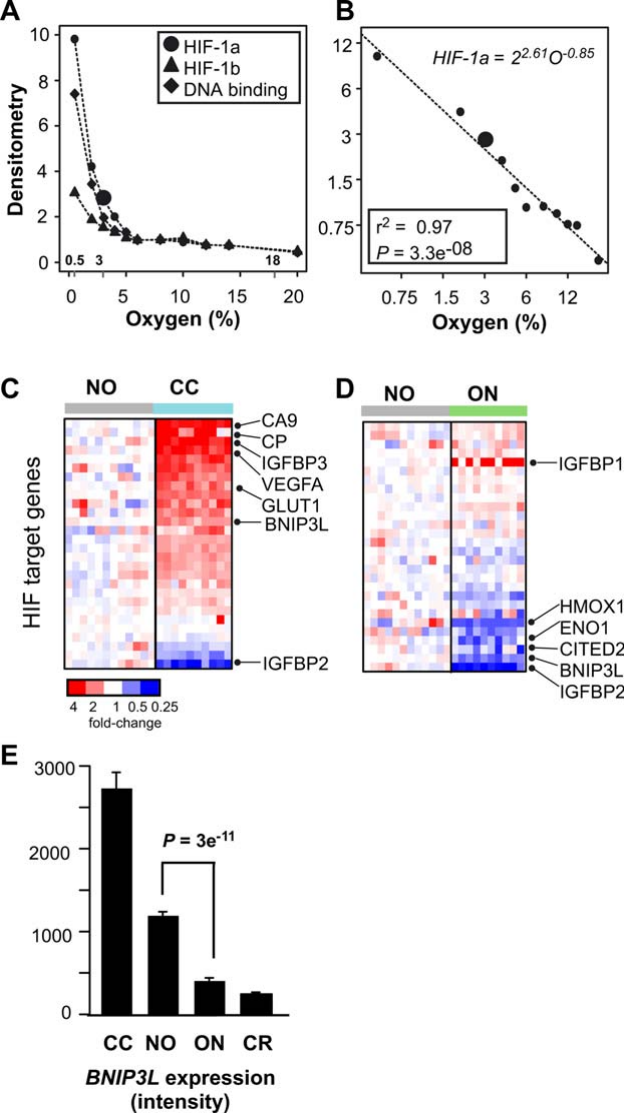
Decreased HIF levels associated with hyperoxic cell state. (A) Normalized densitometry of HIF-1α protein levels (•),HIF-1β protein levels (▴), and HIF-1 DNA-binding activity (⧫) as presented in Figure 5B of the Jiang et al. article. The large (•) highlights the 3% oxygen HIF-1α protein levels in both figures. (B) The densitometry data and oxygen-concentration data presented in (A) were log_2_-transformed and re-plotted. The summary statistics of the best-fit line are also shown. (C,D) Relative gene expression levels of HIF target genes in clear cell RCC (*n* = 10) and renal oncocytoma (*n* = 10) compared with non-diseased kidney (*n* = 12). For each gene, red indicates increased expression, blue decreased expression. (E) Relative expression of *BNI3PL* as determined by gene expression profiling.

In clear cell RCC, an increase in HIFα due to functional inactivation of VHL induces a transcriptional program that mimics cellular exposure to hypoxic conditions. In contrast, in the renal oncocytoma, the functional effects of increased expression of EGLN2 would be to decrease HIFα levels. To examine the cellular effects of decreased HIFα, we re-evaluated previously published data that measured HIF-1 DNA-binding activity, HIF-1α protein levels, and HIF-1β protein levels in cells exposed to hypo- and hyper-oxygenated conditions [6]. Normoxic conditions in the kidney cortex is estimated to be 3–5% oxygen [6]. Induction of a hypo-oxygenated condition was associated with a significant increase in HIFα and HIF activity levels (Figure 4A). Specifically, a six-fold decrease in oxygen concentration (3% to 0.5% oxygen) resulted in approximately a four-fold increase in HIF-1α levels (2.5 to 9.8 densitometry units). Further, we noted that HIF-1α levels change in an analogous manner upon induction of hyper-oxygenated conditions: a six-fold increase in oxygen concentration (3% to 18% oxygen) results in greater than a three-fold decrease in HIF-1α levels (2.5 to 0.75 densitometry units). The association between decreased HIF-1α and hyper-oxygenated conditions is easier to evaluate if the HIF dose-response data is plotted on a log-log scale rather than a linear-linear scale (Figure 4B). The log-log transformed data follow a straight line, indicating that HIFα level and oxygen concentration follow a power-law relationship (i.e., *f(x)* = *ax^k^*), rather than an exponential relationship (i.e., *f(x)* = *ka^x^*). The biological implications of the power-law relationship is that an *n*-fold change in oxygen concentration leads to a proportional *n*-fold change in HIF-1α levels and HIF activity (Figure S3). Moreover, these results demonstrate that while increases in HIF-1α are associated with hypo-oxygenated conditions, decreases in HIF-1α are associated with hyper-oxygenated conditions.

**Figure 5:**
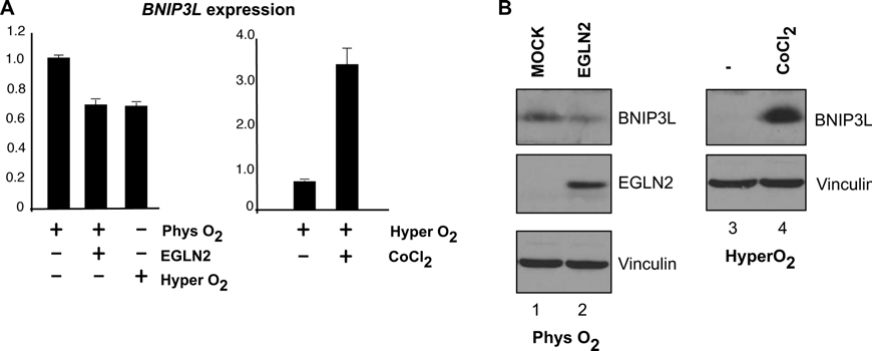
BNI3PL is regulated by an EGLN2 mediated oxygen-sensitive response. (A) *BNIP3L* expression level was measured by qRT-PCR in U2OS cells transfected with plasmids encoding EGLN2 or empty plasmid (MOCK) under physiologic hypoxia (cyclical 0–7% oxygen) or hyper-oxygenated condition (21% oxygen) (left panel). *BNIP3L* expression level was measured by qRT-PCR in U2OS cells maintained under hyper-oxygenated condition with or without CoCl_2_ (right panel). BNIP3L expression was normalized to beta-actin and expression in the MOCK transfected cells was arbitrarily set to 1. Error bars represent the standard deviation between the normalized value versus the MOCK performed in triplicate. (B) The experiment was performed in CAKI cells that contain detectable levels of BNIP3L via Western blot analysis. CAKI cells were transfected with plasmids encoding EGLN2 or empty plasmid (MOCK), lysed, equal amounts of cell lysates separated on SDS-PAGE and immunoblotted with the indicated antibodies (left panel). CAKI cells grown in hyper-oxygenated conditions were treated with or without CoCl_2_. Equal amounts of cell lysates were resolved by SDS-PAGE and immunoblotted with the indicated antibodies (right panel).

To determine whether EGLN2 overexpression is inducing a HIF-mediated hyperoxic cell response in the renal oncocytoma cells, the expression pattern of several known HIF target genes were examined in the renal oncocytoma cells and, for comparison, in clear cell RCC [33]. Consistent with VHL defects present in the clear cell RCC, gene set enrichment analysis revealed a significant up-regulation of the HIF-1 target genes in clear cell RCC (*P* = 0.0001; Figure 4C). Notable up-regulated genes included carbonic anhydrase IX (*CA9*), ferroxidase (*CP*), vascular endothelial growth factor A (*VEGFA*), and glucose transporter (*GLUT1*). However, in the renal oncocytoma cells, a distinct population of HIF-target genes were significantly down-regulated (*P* = 0.01; Figure 4D). Specifically, the HIF-target genes heme oxygenase 1 (*HMOX1*), enolase 1 (*ENO1*), and Cbp/p300-interacting transactivator (*CITED2*) were significantly down-regulated, but genes such as *CA9*, *VEGFA*, and *GLUT1* were not. In addition, the recently identified tumor suppressor *BNIP3L* is downregulated three-fold in the renal oncocytoma cells (Figure 4E). *BNIP3L* is an oxygen-regulated member of the Bcl-2 family (Figure S4). BNIP3L is a pro-death gene (induces features of apoptosis, necrosis and autophagy) and knockdown of this gene is sufficient to convert non-tumorigenic cell lines into tumorigenic lines in xenograft studies [34]–[36]. In support, while hypoxia mimetic treatment significantly induced the expression of *BNIP3L*, *HMOX1*, *ENO1*, and *CITED2* (Figure 5A, right panel and Figure S5), ectopic transient expression of *EGLN2* under physiologic hypoxia (cyclical 0–7% oxygen exposure [37]) was associated with reduced level of expression of these genes in comparison to cells transfected with empty plasmid (Figure 5 and Figure S5). These results demonstrate that over expression of EGLN2 can downregulate HIF1 responsive factors, such as BNIP3L. Moreover, while up-regulation of HIF-target genes such as *VEGFA* are associated with the development of clear cell RCC, these results suggest that down-regulation of distinct subset of HIF-target genes are associated with the development of renal oncocytomas.

## Discussion

A proper oxygen-sensing response is vital to the maintenance of normal cellular functions. Deregulation of HIF, the principal driver of the adaptive response to hypoxia, is associated with the pathogenesis of several diseases, including cancer. While the hypoxic tumor microenvironment - by the virtue of the ubiquitous oxygen-sensing pathway - results in modulation of HIF activity, loss-of-function mutations in a growing list of tumor suppressor genes also can affect HIF function. Mutations in PTEN, PML, TSC, and VHL have been identified in tumor cells that result in the deregulation of HIF via multiple distinct mechanisms involving Akt/PI3K, mTOR and the ubiquitin pathway. Emerging evidence now implicates cancer-causing mutations that directly impinge on EGLNs. For example, mutations in *succinate dehydrogenase* (*SDH*) result in the cytosolic accumulation of succinate, which inhibits EGLNs, leading to the stabilization and activation of HIF-1α [38],[39]. Inactivating germline mutations in EGLN1 have been identified to cause erythrocytosis [13],[14] and deregulation of EGLN3 has been linked to the development of pheochromocytoma, a neuroendocrine tumor of the adrenal glands [15].

In this study, we reveal somatic pairing of chr 19q as a recurrent cytogenetic abnormality in renal oncocytoma that results in dramatic changes in transcription within the paired region. The functional consequence of chromosome joining is formally unknown but it is may disrupt chromatin structure causing the juxtaposition of *cis* and *trans* regulatory regions that modulate the transcription of a large set of genes. The identification of *EGLN2* as a significantly deregulated gene that maps within the paired chr 19q region directly implicates defects in the oxygen-sensing network to the pathobiology of renal oncocytoma. These results suggest that in addition to numerical and structural chromosomal abnormalities, somatic pairing should be considered as a chromosomal event that associates with tumorigenesis.

Although the loss of EGLN2 does not lead to decreased HIF1α accumulation, perhaps due to the compensatory activity of EGLN3, the data from this study suggest that overexpression of ELGN2 leads to decreased HIF1 levels. More recently, an E3 ubiquitin ligase called Siah2 was identified to target EGLN2 for ubiquitin-mediated destruction and thereby revealing another level of HIF regulation [40]. The activity of Siah2 is induced under physiologic hypoxia (<10% oxygen), resulting in reduced levels of EGLN2 and stabilization of HIF-1α. The present findings suggest that the overexpression of EGLN2 via somatic pairing is sufficient to counteract the suppressive activity of Siah2 under physiologic hypoxia. Under hyper-oxygenated conditions (21% oxygen; frequently used as experimental normoxia), Siah2 activity is attenuated via a yet-defined mechanism, resulting in the increased abundance of EGLN2 and concomitant reduction in the level of HIF-1α [40]. The ectopic expression of EGLN2 under 21% oxygen did not result in further diminution of HIF-target gene expression (data not shown), which is likely due to the fact that endogenous EGLN2 is highly abundant or that every available EGLN2 is already activated under hyper-oxygenated conditions.

HIF-regulated genes are involved in many physiological processes including angiogenesis, metabolism, cell proliferation, survival, and apoptosis. As such, disruption in the regulation of HIF may affect several regulatory pathways that contribute to the transformation of normal cells into cancer cells. Evasion of apoptosis is one of the hallmark features of cancer cells and represents a key oncogenic event. BNIP3L is a regulator of p53-dependent apoptosis and silencing of BNIP3L has been associated with enhanced tumorgenicity and reduced apoptotic response [36]. We show here that BNIP3L is one of several HIF-responsive genes governed, in part, by EGLN2. Therefore, we propose that the downregulation of BNIP3L is the result of chromosome-pairing induced upregulation of EGLN2 and that downregulation of BNIP3L contributes to the inhibition of apoptosis to facilitate oncocytoma cell survival and growth.

The disruption of HIF activity has been associated with kidney cancer related to VHL disease, sporadic clear cell RCC, and hereditary papillary RCC [38],[41],[42]. The present study reveals deregulation of the oxygen-sensing response in renal oncocytoma, as well as chromophobe RCCs (which display DNA amplification mediated up-regulation of EGLN2) and thereby supporting the dysfunction of HIF pathway as a common and perhaps central theme in the pathogenesis of kidney cancer.

## Materials and Methods

### Gene Expression Profiling and Analysis

Single-color expression profiles were generated using the HG-U133 Plus 2.0™ chipset (Affymetrix, Santa Clara, CA) from renal oncocytoma (*n* = 10), chromophobe RCC (*n* = 10), and nondiseased kidney (*n* = 12) samples as described [43]. The gene expression data can be obtained at the Gene Expression Omnibus (GSE8271 and GSE7023). Analysis was performed using BioConductor version 2.0 software. Data preprocessing was performed using the RMA method as implemented in the *affy* package and using updated probe set mappings such that a single probe set describes each gene [44],[45],[46]. Chromosomal abnormalities were predicted using the comparative genomic microarray analysis (CGMA) method as implemented in the *reb* package [47]. Briefly, for each measured gene, the gene expression value was normalized such that the average gene expression value in the nondiseased samples was subtracted from the tumor-derived gene expression value. A Welsh's *t*-test was applied to the relative gene expression values that mapped to each chromosome arm. For the smoothing curve, the normalized expression values derived from genes mapping to chromosome 19 were replaced by a summary score that comprised a running two-sided *t*-test statistic using window sizes of 61, 245, and 611 (representing 5%, 20%, and 50% of the length of the chromosome). The results of the three smoothing curves were averaged. To identify HIF-interacting genes, the Entrez Gene database (http://www.ncbi.nlm.nih.gov/sites/entrez) was searched using the search string ‘(“HIF” or “VHL”) and “19”[chr] and “homo sapiens”[orgn]’. Differentially expressed genes were identified using a two-sided *t*-test. For HIF target gene analysis, 36 known HIF-responsive genes identified in Maynard et al. were isolated [33]. Enrichment of up- and down-regulated genes in the HIF target gene set was performed by comparing differences in the expression level ranks between HIF target gene set to the results of 10,000 randomly generated 36-gene sets. Ranks were based on tumor versus normal expression comparisons as implemented in the *limma* package [48].

### DNA Copy Number Profiling and Analysis

SNP allele calls were generated using the GeneChip Mapping 100 K Set™ (Affymetrix, Santa Clara, CA) according to the manufacturer's supplied protocol. Image quantification was performed with a GeneChip Scanner 3000 and the resulting data was processed using GCOS 1.4 (Affymetrix, Santa Clara, CA) with default analysis settings. Allele calls were generated using GTYPE 4.0 (Affymetrix, Santa Clara, CA) with a confidence threshold set at 0.25. Raw copy numbers in log_2_-transformed format (non-paired reference and test samples) were exported from the CNAG version 2.0 (Affymetrix, Santa Clara, CA) software using normal references downloaded from Affymetrix (http://www.affymetrix.comccnt_reference_data). DNA copy number changes were visualized by data smoothing in which raw copy number values were replaced by a summary score that comprised a running 1-sided *t*-test statistic with window size set to 31, where each SNP probe along with 15 5′ SNPs and 15 3′ SNPs were included in the window. DNA copy number data can be obtained at the Gene Expression Omnibus (GSE8271).

### FISH and WCP

Bacterial artificial chromosomes (BACs) RP11-157B13 (19p12), RP11-1137G4 (19p13.3), RP11-15A1 (19q13.31) were obtained from the Children's Hospital Oakland Research Institute (http://bacpac.chori.org) and BAC CTC-429C10 (19q13.41) was purchased from Invitrogen (Invitrogen Corporation, Carlsbad, CA). These clones were labeled with either SpectrumGreen or SpectrumOrange (Abbott Molecular Inc, Des Plaines, IL) by nick translation and applied to tissue touch preps of oncocytoma samples as described [49], with the exception that slides were counterstained with VECTASHIELD (Vector Laboratories, Inc. Burlingame, CA) anti-fade 4′,6-diamidino-2-phenylindole (DAPI). Telomere-specific DNA probes, the chr 1,5,19 alpha satellite probe, and the arm-specific paints were purchased from Q-BIOgene (MP Biomedicals, Solon, OH). FISH was performed using these probes according to the manufacturer's supplied protocol. As the alpha satellite probe cross-hybridizes to chromosome 1 and chromosome 5, in all studies chromosome 19 was co-labeled with a probe that maps distal to the centromere, RP11-157B13 (19p12). In addition, analysis of the centromeric probe on the metaphase spreads of control cells revealed that hybridization to chromosome 1 resulted in a significantly brighter signal (data not shown). These hybridization characteristics allowed the discrimination between chr 1 and 5 cross-hybridization.

### Image Analysis

For image quantification, three separate photomicrographs containing five, six, and three cells, respectively, in which the 19q31.31 FISH signals were in the same image plane were obtained. Photomicrographs were processed using the *rtiff* package for the R environment [50]. The fluorescent FISH signals were automatically segmented from background using the method of Ridler and Calvard [51], individual spots were identified using the connected component algorithm [52], and the number of pixels per feature were calculated. Twelve doublet FISH signals and eight singlet FISH signals were compared. Differences in size were evaluated using a one-sided Student's *t*-test.

### Cells

U2OS osteosarcoma cell and CAKI renal clear-cell carcinoma cell lines were obtained from the American Type Culture Collection (Rockville, MD) and maintained in Dulbecco's modified Eagle's medium supplemented with 10% heat-inactivated fetal bovine serum (Sigma, Milwaukee, WI) at 37°C in a humidified 5% CO_2_ atmosphere. Cyclic hypoxia treatment of cells were performed in humidified chambers at 37°C and flushed with 5% CO_2_ balance N_2_ for 30 min, followed by 5% CO_2_ and 7% O_2_ balance N_2_ for 30 min as one cycle. Cells were grown in these chambers for 16 hours [53].

### Antibodies

Polyclonal anti-EGLN2 and anti-BNIP3L antibodies were obtained from Bethyl Laboratories (Montgomery, TX) and Sigma (Milwaukee, WI), respectively. Polyclonal HIF1α and monoclonal HIF2α antibodies were obtained from BD Biosciences (San Jose, CA) and Novus (Littleton, CO), respectively. Monoclonal anti-vinculin antibody was obtained from Abcam (Cambridge, MA).

### Plasmids

Mammalian expression plasmids pcEglN2 was generated by PCR from Flag-EglN2, a kind gift from Dr. Mircea Ivan, using primers 5′-GACGACGGATCCATGGACAGCCCGTGCCAGC-3′ and 5′-GACGACGAATTCCTAGGTGGGCGTAGGCGGC -3′. The PCR product was then ligated into the *Bam*HI and *Eco*RI sites in pcDNA3(+). Plasmid was confirmed by direct DNA sequencing.

### Immunoblotting

Western blotting were performed as described previously [54].

### Quantitative Real-Time PCR

For first-strand cDNA synthesis, 1 µl of oligo(dT)_23_ primer (Sigma) was incubated with 5 µg of RNA and distilled H_2_O (total reaction volume of 20 µl) for 10 min at 70°C in a thermal cycler (MJ Research, Boston, MA). The mixture was cooled to 4°C, at which time 4 µl of 5× first-strand reaction buffer, 2 µl of 0.1 M DTT, 1 µl of a 10 mM concentration of each deoxynucleoside triphosphate, and 1 µl of Superscript II reverse transcriptase (Invitrogen) were added. cDNA synthesis was performed for 1.5 h at 42°C, followed by 15 min at 70°C in the thermal cycler. Human genomic DNA standards (human genomic DNA was obtained from Roche, Mannheim, Germany) or cDNA equivalent to 20 ng of total RNA were added to the quantitative PCR (qPCR) reaction mixture in a final volume of 10 µl containing 1× PCR buffer (without MgCl_2_), 3 mM MgCl_2_, 0.25 units of Platinum *Taq* DNA polymerase, a 0.2 mM concentration of each deoxynucleoside triphosphate, 0.3 µl of SYBR Green I, 0.2 µl of ROX reference dye, and a 0.5 µM concentration of each primer (Invitrogen). Amplification conditions were as follows: 95°C (3 min), 40 cycles of 95°C (10 s), 65°C (15 s), 72°C (20 s), and 95°C (15 s). qPCR was performed using the ABI Prism 7900HT Sequence Detection System (Applied Biosystems, Foster City, CA). Gene-specific oligonucleotide primers designed using Primer Express (Applied Biosystems) were as follows: *BNIP3L* primer set (5′- CTGCACAAACTTGCACATTG-3′ and 5′- TAATTTCCACAACGGGTTCA-3′), *HMOX1* primer set (5′-GAATTCTCTTGGCTGGCTTC-3′ and 5′- TCCTTCCTCCTTTCCAGAGA-3′), *ENO1* primer set (5′- CAGCTCTAGCTTTGCAGTCG-3′ and 5′-GACACGAGGCTCACATGACT-3′), *CITED2* primer set (5′-ACTGCACAAACTGCCATCTC-3′ and 5′-CAGCCAACTTGAAAGTGAACA-3′), *beta-actin* primer set (5′- GGATCGGCGGCTCCAT-3′ and 5′- CATACTCCTGCTTGCTGATCCA-3′), *GLUT-1* primer set (5′- CACCACCTCACTCCTGTTACTT-3′ and 5′-CAAGCATTTCAAAACCATGTTTCTA-3′). SYBR Green I fluoresces during each cycle of the qPCR by an amount proportional to the quantity of amplified cDNA (the amplicon) present at that time. The point at which the fluorescent signal is statistically significant above background is defined as the cycle threshold (*C_T_*). Expression levels of the various transcripts were determined by taking the average *C_T_* value for each cDNA sample performed in triplicate and measured against a standard plot of *C_T_* values from amplification of serially diluted human genomic DNA standards. Since the *C_T_* value is inversely proportional to the log of the initial copy number, the copy number of an experimental mRNA can be obtained from linear regression of the standard curve. A measure of the relative difference in copy number was determined for each mRNA. Values were normalized to expression of *beta-actin* mRNA and represented as the mean value experiments performed in triplicate±standard deviations.

### Purification of HIF Prolyl Hydroxylase 1 (EGLN2/PHD1)

Extracts containing enriched EGLN2 were purified from rabbit reticulocyte lysate as previously described [8]. Briefly, approximately 1 L of rabbit reticulocyte lysate (Green Hectares, Oregon, WI) was diluted to 5 L in 50 mM Tris-HCl (pH 7.4), 0.1 M KCl, and 5% (vol/vol) glycerol and then was precipitated with 0.213 g/ml (NH_4_)_2_SO_4_. After centrifugation at 16,000×*g* for 45 min at 4°C, the resulting supernatant was precipitated with an additional 0.153 g/ml (NH_4_)_2_SO_4_. After centrifugation at 16,000×*g* for 45 min at 4°C, the pellet was resuspended in Buffer A (40 mM HEPES-NaOH [pH 7.4] and 5% (vol/vol) glycerol), dialyzed against Buffer A to a conductivity equivalent to Buffer A containing 0.2 M KCl, and applied at 0.5 L/h to a 0.5 L phosphocellulose (Whatman, P11) column equilibrated in Buffer A containing 0.2 M KCl. The phosphocellulose column was eluted stepwise at 1 L/h with Buffer A containing 0.5 M KCl, and 100-ml fractions were collected. Proteins eluting in the phosphocellulose 0.5 KCl step were pooled and precipitated with 0.4 g/ml (NH_4_)_2_SO_4_. After centrifugation at 16,000×*g* for 45 min at 4°C, the pellet was resuspended in 4 ml of Buffer A. Following centrifugation at 35,000×*g* for 30 min at 4°C, the resulting supernatant was applied at 2 ml/min to a TSK SW3000 HPLC column (Toso-Haas, Montgomeryville, PA; 21.5×600 mm) equilibrated in Buffer A containing 0.15 M KCl. The SW3000 column was eluted at 2 ml/min, and 4 ml fractions containing enriched EGLN2 were collected.

### In Vitro Binding Assay

An *in vitro* binding assay was performed as described previously [3]. TNT reticulocyte lysate (Promega) translation products were synthesized in the presence or absence of ^35^S-methionine. HIF1α-(ODD) translation products were incubated with cellular extract fractions containing enriched EGLN2, where indicated, for 30 min at 37°C. Gal4-HA-HIF-1α (10 µl) and HA-VHL (10 µl) translation products were incubated with the indicated antibodies and protein A-Sepharose in 750 µl of EBC buffer (50 mM Tris [pH 8], 120 mM NaCl, 0.5% Nonidet P-40). After five washes with NETN buffer (20 mM Tris (pH 8), 100 mM NaCl, 0.5% Nonidet P-40, 1 mM EDTA), the bound proteins were resolved on SDS-PAGE and detected by autoradiography.

### In Vitro Ubiquitylation Assay

An *in vitro* ubiquitylation assay was performed as described previously [3]. ^[35S]^Methionine-labeled reticulocyte lysate Gal4-HA-HIF1α(ODD) (4 µl) were incubated in RCC 786-O S100 extracts (100–150 µg). Reactions were supplemented with an increasing titration of EGLN2-enriched cellular fraction where indicated. Additional reaction supplements include 8 µg/µl ubiquitin (Sigma), 100 ng/µl ubiquitin-aldehyde (BostonBiochem, Inc., Cambridge, MA), and an ATP-regenerating system (20 mM Tris [pH 7.4], 2 mM ATP, 5 mM MgCl_2_, 40 mM creatine phosphate, 0.5 µg/µl of creatine kinase) in a reaction volume of 20–30 µl for 1.5 h at 30°C.

### HIF Dose Response

Figure 5B from the Jiang et al. article [6] was obtained in Portable Document Format (PDF, Adobe Systems), imported into Canvas 9 (ACD Systems), and the x- and y-graphic device coordinates of each data point, the x-axis ticks (oxygen concentration), and the y-axis ticks (densitometry) were extracted. Linear interpolation was used to convert the graphic device coordinates to protein densitometry measurements and oxygen concentrations. Based on comparisons between the extracted oxygen concentrations (0.5, 1.9, 2.9, 3.9, 4.8, 5.8, 7.9, 9.9, 11.9, 13.9, 19.9) and the actual oxygen concentrations (0.5, 2, 3, 4, 5, 6, 8, 10, 12, 14, 20), the extracted data varied on average less than 2% from the original data. The densitometry and oxygen concentration data were log_2_-transformed and linear model fit to the transformed data. The best-fit power-law equation is *HIF1α* = *2^2.61^O^−0.85^*, where *HIF1α* represents HIF-1α protein levels and *O* represent oxygen concentration.

## Supporting Information

Figure S1 Regional transcriptional abnormalities in renal tumors.
(0.06 MB PDF)

Figure S2 Expression of EGLN2 in Chromophobe RCC.
(0.22 MB PDF)

Figure S3 HIF1 protein and HIF1 DNA-binding activity levels in response to changing oxygen concentration.
(0.22 MB PDF)

Figure S4 BNIP3L expression associates with HIF expression.
(0.15 MB PDF)

Figure S5 Hypoxia-responsive genes repressed in oncocytoma are suppressed by EGLN2.
(0.01 MB PDF)

Table S1 Chromosome 19 FISH patterns in chromophobe RCC.
(0.04 MB PDF)

Table S2 Chromosome 19 FISH patterns in oncocytoma.
(0.04 MB PDF)

Table S3 Cancer related genes mapping to chromosome 19q.
(0.07 MB PDF)
