# Correction: Somatic Pairing of Chromosome 19 in Renal Oncocytoma Is Associated with Deregulated EGLN2-Mediated Oxygen-Sensing Response

**DOI:** 10.1371/annotation/7f57058f-cc02-45a6-bfab-a835619e3916

**Published:** 2008-09-18

**Authors:** Julie M. Koeman, Ryan C. Russell, Min-Han Tan, David Petillo, Michael Westphal, Katherine Koelzer, Julie L. Metcalf, Zhongfa Zhang, Daisuke Matsuda, Karl J. Dykema, Heather L. Houseman, Eric J. Kort, Laura L. Furge, Richard J. Kahnoski, Stéphane Richard, Annick Vieillefond, Pamela J. Swiatek, Bin Tean Teh, Michael Ohh, Kyle A. Furge

The protein name EGLN2 was misspelled in the article title and citation, as well as in the Abstract and Discussion sections, as noted on the article. In all of these cases, ELGN2 should be EGLN2. The correct article title is: Somatic Pairing of Chromosome 19 in Renal Oncocytoma Is Associated with Deregulated EGLN2-Mediated Oxygen-Sensing Response. The correct citation is: Koeman JM, Russell RC, Tan M-H, Petillo D, Westphal M, et al. (2008) Somatic Pairing of Chromosome 19 in Renal Oncocytoma Is Associated with Deregulated EGLN2-Mediated Oxygen-Sensing Response. PLoS Genet 4(9): e1000176. doi:10.1371/journal.pgen.1000176.

