# Supplementary material for: Somatic Pairing of Chromosome 19 in Renal Oncocytoma Is Associated with Deregulated ELGN2-Mediated Oxygen-Sensing Response
Source: PLoS Genet. 2008 Sep 5;4(9):e1000176. doi: 10.1371/journal.pgen.1000176 (PMC2518213; doi:10.1371/journal.pgen.1000176)
Supplement: Figure S1 — Regional transcriptional abnormalities in renal tumors. (0.06 MB PDF) [file pgen.1000176.s001.pdf]

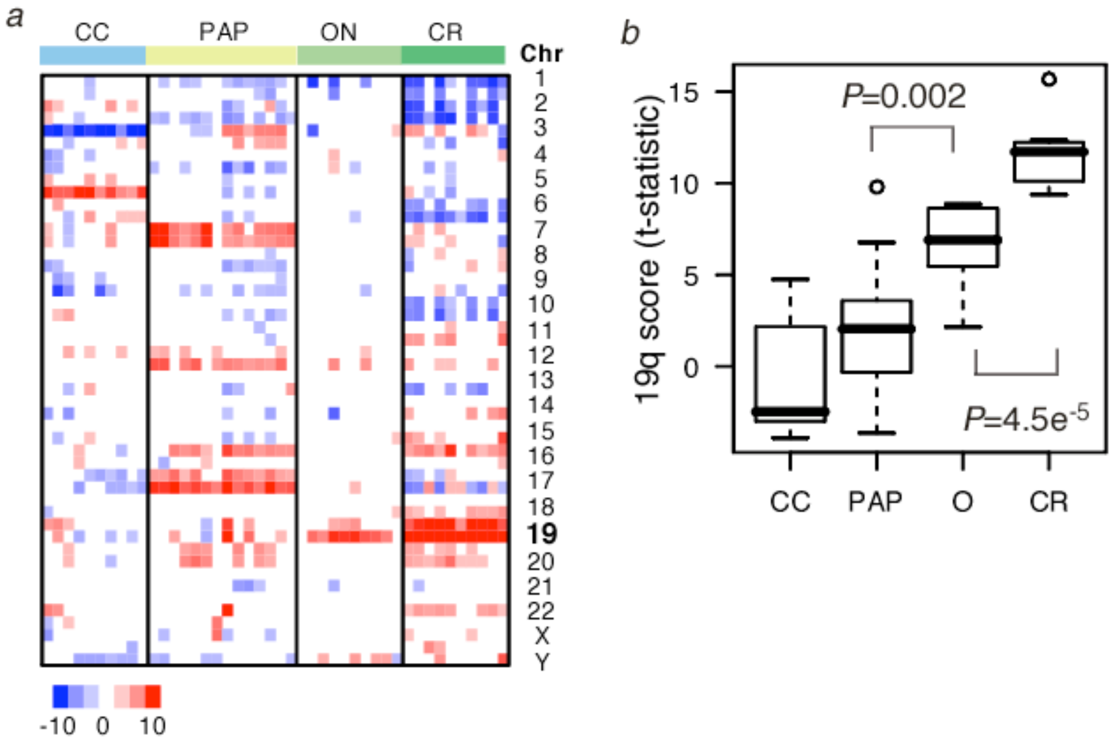

Figure S1. Regional transcriptional abnormalities in renal tumours. **a,** Regional transcriptional abnormalities were computed using the CGMA method as described in the Material and Methods. Shown are the resulting summary scores (t-statistics) from gene expression profiles derived from oncocytoma (ON, n=10), chromophobe RCC, (CR, n=10), clear cell RCC (CC,n=10) and papillary RCC (PAP, n=14). Genomic regions that have higher (red) or lower (blue) overall gene expression when compared to non-diseased tissues are shown. Only the most significant summary scores are shown ($P <$ 0.005). **b,** The complete set of summary statistics (t-statistics) for the chromosome 19q region were partitioned based on tumor subtype and plotted. Significant differences in summary scores are shown.
