# Supplementary material for: Somatic Pairing of Chromosome 19 in Renal Oncocytoma Is Associated with Deregulated ELGN2-Mediated Oxygen-Sensing Response
Source: PLoS Genet. 2008 Sep 5;4(9):e1000176. doi: 10.1371/journal.pgen.1000176 (PMC2518213; doi:10.1371/journal.pgen.1000176)
Supplement: Figure S2 — Expression of EGLN2 in Chromophobe RCC. (0.22 MB PDF) [file pgen.1000176.s002.pdf]

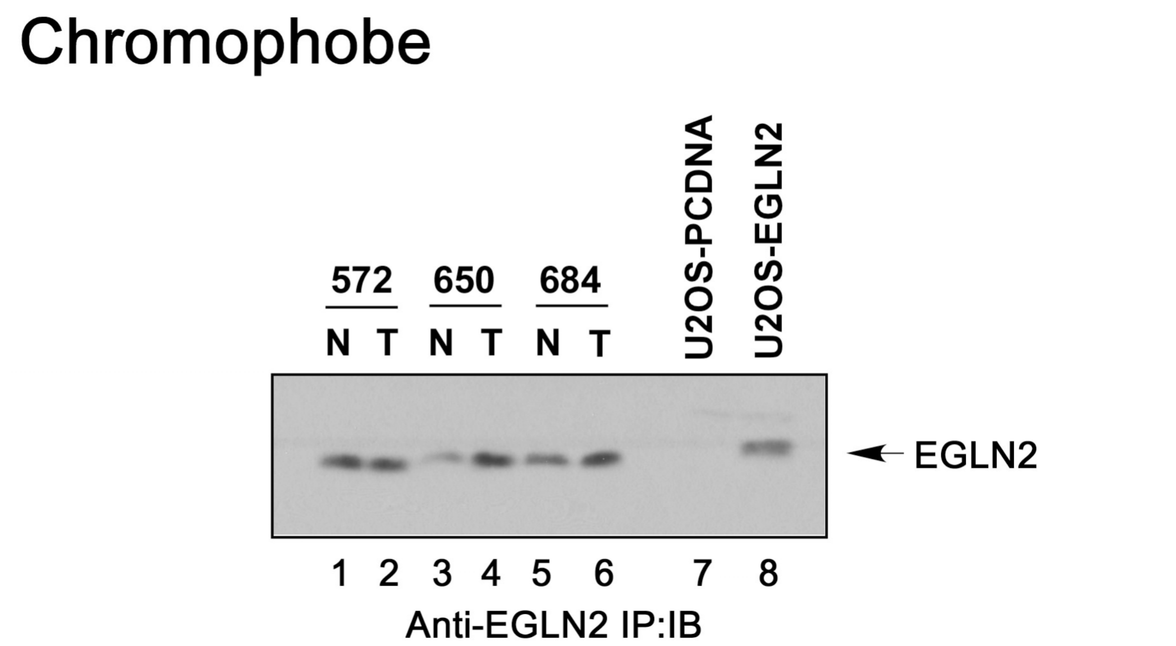


**Figure S2. Expression of EGLN2 in Chromophobe RCC**. Anti-EGLN2 immunoblot analysis of ELGN2 immunoprecipitated from whole-cell extracts prepared from chromophobe RCC or patient-matched normal tissue samples (lanes 1-6) and exogenously expressed EGLN2 controls (lanes 7-8). U2OS were transfected with plasmid containing human EGLN2 or empty vector alone (PCDNA); EGLN2 appears as a single band of 45 kDa
