# Supplementary material for: Somatic Pairing of Chromosome 19 in Renal Oncocytoma Is Associated with Deregulated ELGN2-Mediated Oxygen-Sensing Response
Source: PLoS Genet. 2008 Sep 5;4(9):e1000176. doi: 10.1371/journal.pgen.1000176 (PMC2518213; doi:10.1371/journal.pgen.1000176)
Supplement: Figure S3 — HIF1 protein and HIF1 DNA-binding activity levels in response to changing oxygen concentration. (0.22 MB PDF) [file pgen.1000176.s003.pdf]

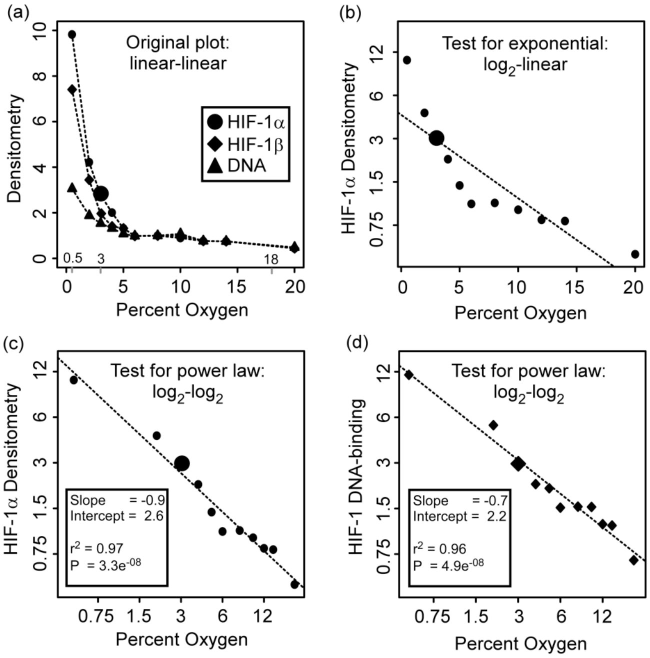

Figure S3. HIF1αprotein and HIF1 DNA-binding activity levels in response to changing oxygen concentration. Figure 5B from the Jiang, et al. article was obtained in Portable Document Format (PDF, Adobe Systems), imported into Canvas 9 (ACD Systems), and the *x*- and *y*-graphic device coordinates of each data point was extracted. Linear interpolation was used to convert the graphic device coordinates to densitometry measurements (R 2.5.0). To ensure the data were extracted accurately, the *x*-axis ticks were also extracted and compared to the original *x*-axis ticks. The *x*-axis measurement error was on average 2% and ranged between 0% and 5%. (a) Reproduction of Figure 5B presented in the original Jiang et al. study. Briefly, this figure represents normalized densitometry of HIF1α protein levels (●), HIF1β protein levels (▲), and HIF1 DNA-binding activity (◆) in HeLa cells. (b) The densitometry data presented in (a) were $\log_2$-transformed and re-plotted. The best fit line is also shown (c) The densitometry and oxygen concentration data presented in (a) were $\log_2$-transformed and re-plotted. Summary statistics of a linear model fit to the $\log_2$-transformed data are also shown. The best fit equation is $HIF1\alpha = 2^{2.61}O^{-0.85}$. (d) Power-law fit of the HIF1 DNA-binding activity. The corresponding power-law equation is $HIF1_{dna} = 2^{2.19}O^{-0.74}$
