# Supplementary material for: Somatic Pairing of Chromosome 19 in Renal Oncocytoma Is Associated with Deregulated ELGN2-Mediated Oxygen-Sensing Response
Source: PLoS Genet. 2008 Sep 5;4(9):e1000176. doi: 10.1371/journal.pgen.1000176 (PMC2518213; doi:10.1371/journal.pgen.1000176)
Supplement: Figure S4 — BNIP3L expression associates with HIF expression. (0.15 MB PDF) [file pgen.1000176.s004.pdf]

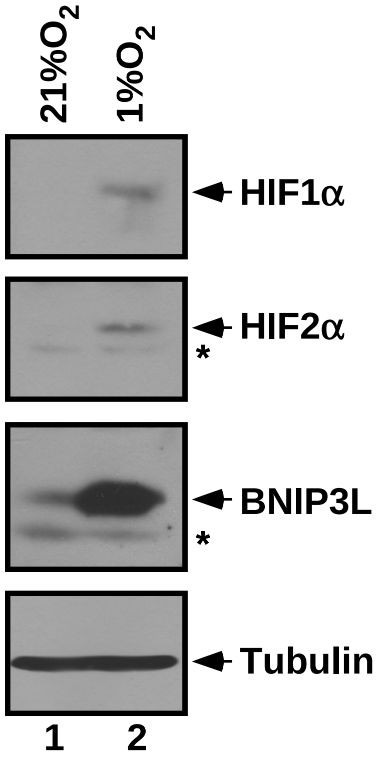

Figure S4. BNIP3L positively correlates with HIF1α and HIF2α. Whole cell extracts were prepared from U2OS cells maintained under 21% or 1% oxygen and separated by SDS-PAGE and immunoblotted with the indicated antibodies. *denotes non-specific band.
