# Supplementary material for: Somatic Pairing of Chromosome 19 in Renal Oncocytoma Is Associated with Deregulated ELGN2-Mediated Oxygen-Sensing Response
Source: PLoS Genet. 2008 Sep 5;4(9):e1000176. doi: 10.1371/journal.pgen.1000176 (PMC2518213; doi:10.1371/journal.pgen.1000176)
Supplement: Figure S5 — Hypoxia-responsive genes repressed in oncocytoma are suppressed by EGLN2. (0.01 MB PDF) [file pgen.1000176.s005.pdf]

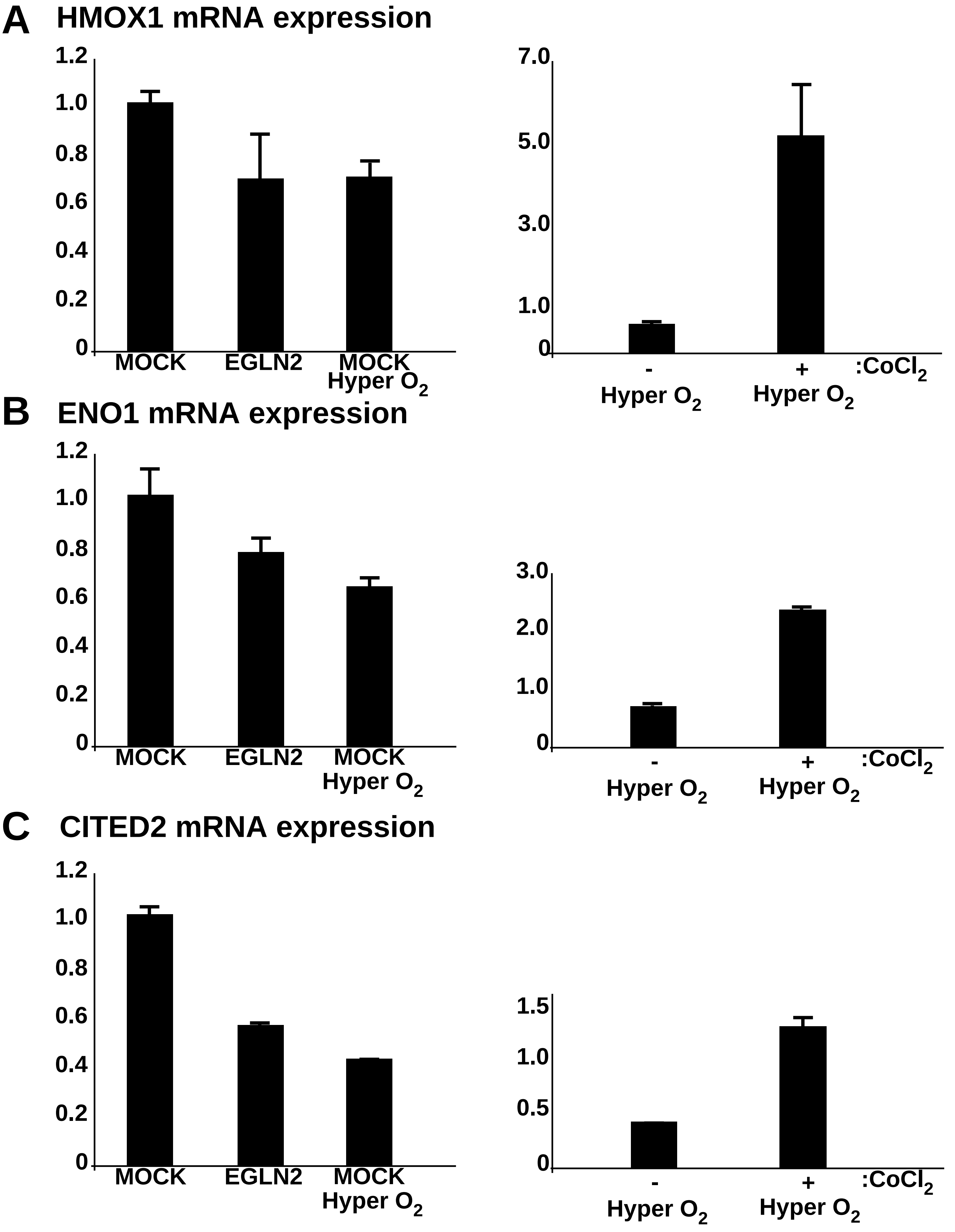

Figure S5. Hypoxia-responsive genes repressed in oncocytoma are suppressed by EGLN2. HMOX1, ENO1 and CITED2 expression was measured by qRT-PCR as described in Figure 5a.
