# Supplementary material for: Somatic Pairing of Chromosome 19 in Renal Oncocytoma Is Associated with Deregulated ELGN2-Mediated Oxygen-Sensing Response
Source: PLoS Genet. 2008 Sep 5;4(9):e1000176. doi: 10.1371/journal.pgen.1000176 (PMC2518213; doi:10.1371/journal.pgen.1000176)
Supplement: Table S1 — Chromosome 19 FISH patterns in chromophobe RCC. (0.04 MB PDF) [file pgen.1000176.s006.pdf]

Table S1. Chromosome 19 FISH patterns[a]

			Signal					Cells
**Cell type**	**Probe**	**Location**	**1***	**1**	**2**	**3+**	**Other**	**Counted**
Kidney	RP11-1137G4	19p13.3	5.5%	5.0%	**88.5%**	2.0%	NC	200
Kidney	RP11-15A1	19q13.31	6.5%	2.5%	**90.0%**	1.0%	NC	200
CR 86	RP11-1137G4	19p13.3	0.0%	0.0%	40.0%	**48.0%**	NC	52
CR 86	RP11-15A1	19q13.31	8.0%	0.0%	40.0%	**48.0%**	NC	52
CR 15	PTEL 19P	19p tel	0.0%	0.0%	13.0%	**87.0%**	NC	100
CR 15	PTEL 19Q	19q tel	0.0%	0.0%	13.0%	**87.0%**	NC	100
CR 9	RP11-37G4	19p13.3	2.0%	4.0%	65.0%	**29.0%**	NC	100
CR 9	RP11-15A1	19q13.31	7.0%	3.0%	58.0%	**31.0%**	NC	100

[a]Tumor touch preps were labeled with the indicated probes and the number of signals per cell were counted. NC indicates not counted. CR indicate chromophobe RCC and numbers indicate samples. Results from normal kidney are also shown (Kidney) An asterisks (*) indicates a large signal or two signals in close proximity
