# Supplementary material for: Somatic Pairing of Chromosome 19 in Renal Oncocytoma Is Associated with Deregulated ELGN2-Mediated Oxygen-Sensing Response
Source: PLoS Genet. 2008 Sep 5;4(9):e1000176. doi: 10.1371/journal.pgen.1000176 (PMC2518213; doi:10.1371/journal.pgen.1000176)
Supplement: Table S2 — Chromosome 19 FISH patterns in oncocytoma. (0.04 MB PDF) [file pgen.1000176.s007.pdf]

Table S2. Chromosome 19 FISH patterns[a]

			Signal					Cells
**Cell type**	**Probe**	**Location**	**1***	**1**	**2**	**3+**	**Other**	**Counted**
Kidney	RP11-1137G4	19p13.3	5.5%	5.0%	**88.5%**	2.0%	NC	200
Kidney	RP11-15A1	19q13.31	6.5%	2.5%	**90.0%**	1.0%	NC	200
ON 05	PTEL 19P	19p tel	0.0%	17.0%	**83.0%**	0.0%	NC	200
ON 05	RP11-1137G4	19p13.3	0.0%	15.0%	**80.0%**	5.0%	NC	100
ON 05	RP11-157B13	19p12	0.0%	2.0%	**98.0%**	0.0%	NC	200
ON 05	19SAT	19 cen	NC	NC	NC	0.0%	NC	NC
ON 05	RP11-15A1	19q13.31	**53.0%**	1.0%	45.9%	0.0%	NC	220
ON 05	CTC-429C10	19q13.41	**28.0%**	0.0%	68.0%	4.0%	NC	53
ON 05	PTEL 19Q	19q tel	**53.5%**	15.0%	29.5%	0.0%	NC	200
ON 19	PTEL 19P	19p tel	7.0%	3.0%	**90.0%**	0.0%	NC	100
ON 19	PTEL 19Q	19q tel	**52.0%**	0.0%	48.0%	0.0%	NC	100
ON 20	PTEL 19P	19p tel	6.0%	3.0%	**91.0%**	0.0%	NC	100
ON 20	PTEL 19Q	19q tel	**52.0%**	0.0%	45.0%	0.0%	NC	100
ON 05	ASP019P-G	19p-arm	NC	0.0%	**84.0%**	NC	16.0%	255
ON 05	ASP019Q-R	19q-arm	NC	**55.0%**	29.0%	NC	16.0%	255
ON 19	ASP019P-G	19p-arm	NC	0.0%	**87.0%**	NC	13.0%	259
ON 19	ASP019Q-R	19q-arm	NC	**57.0%**	30.0%	NC	13.0%	259
ON 20	ASP019P-G	19p-arm	NC	0.0%	**88.5%**	NC	11.5%	255
ON 20	ASP019Q-R	19q-arm	NC	**52.5%**	36.0%	NC	11.5%	255

[a]Tumour touch preps were labeled with the indicated probes and the number of signals per cell were counted. NC indicates not counted. ON and CR indicate oncocytoma and chromopobe RCC samples, respectively. Results from normal kidney are also shown (Kidney) An asterisks (*) indicates a large signal or two signals in close proximity
