# Supplementary material for: Somatic Pairing of Chromosome 19 in Renal Oncocytoma Is Associated with Deregulated ELGN2-Mediated Oxygen-Sensing Response
Source: PLoS Genet. 2008 Sep 5;4(9):e1000176. doi: 10.1371/journal.pgen.1000176 (PMC2518213; doi:10.1371/journal.pgen.1000176)
Supplement: Table S3 — Cancer related genes mapping to chromosome 19q. (0.07 MB PDF) [file pgen.1000176.s008.pdf]

Table S3a. Kidney cancer-related deregulated genes that map to chromosome 19q

Entrez Gene ID	Symbol	Description	Band	Fold-Change	P-value[a]	Kidney Cancer Related[b]
**112398**	**EGLN2**	**egl nine homolog 2 (C. elegans)**	**19q13.2**	**1.16**	**6.49E-03**	**3**
5670	PSG2	pregnancy specific beta-1-glycoprotein 2	19q13.1-q13.2	2.35	1.63E-06	1
5673	PSG5	pregnancy specific beta-1-glycoprotein 5	19q13.2	2.05	3.26E-06	1
201516	ZSCAN4	zinc finger and SCAN domain containing 4	19q13.43	1.37	6.31E-05	1
2067	ERCC1	excision repair cross-complementing	19q13.2-q13.3	1.4	9.79E-04	1
898	CCNE1	cyclin E1	19q12	1.28	1.16E-03	1
3802	KIR2DL1	killer cell immunoglobulin-like receptor	19q13.4	1.28	2.46E-03	1
27036	SIGLEC7	sialic acid binding Ig-like lectin 7	19q13.3	1.23	2.89E-03	1
5671	PSG3	pregnancy specific beta-1-glycoprotein 3	19q13.2	1.41	4.43E-03	1
11284	PNKP	polynucleotide kinase 3'-phosphatase	19q13.3-q13.4	1.27	4.68E-03	1
558	AXL	AXL receptor tyrosine kinase	19q13.1	1.2	4.89E-03	1

[a]Genes that mapped to chromosome 19q were examined for differential expression between renal oncocytoma and non-diseased kidney as as described in the Materials and Methods. Shown are adjusted significance values determined using the false discovery rate (FDR) method
[b,c]For each gene, the PubMed database was searched to identify publications that referenced the gene. For each publication, the Medical Subject Headings (MeSH) terms were searched for references to [c]cancer or [d]specifically to renal/kidney cancer.

Table S3b. Cancer-related deregulated genes that map to chromosome 19q

Entrez Gene ID	Symbol	Description	Band	Fold-Change	P-value[a]	Cancer Related[c]
354	KLK3	kallikrein-related peptidase 3	19q13.41	1.66	7.01E-05	35
898	CCNE1	cyclin E1	19q12	1.28	1.16E-03	34
2068	ERCC2	excision repair cross-complementing rodent repai	19q13.3	1.28	4.32E-04	21
27113	BBC3	BCL2 binding component 3	19q13.3-q13.4	1.41	7.78E-03	19
2067	ERCC1	excision repair cross-complementing rodent repai	19q13.2-q13.3	1.4	9.79E-04	12
558	AXL	AXL receptor tyrosine kinase	19q13.1	1.2	4.89E-03	11
2821	GPI	glucose phosphate isomerase	19q13.1	1.66	6.05E-05	10
5655	KLK10	kallikrein-related peptidase 10	19q13.3-q13.4	1.76	1.73E-04	8
5817	PVR	poliovirus receptor	19q13.2	1.19	6.58E-04	8
9622	KLK4	kallikrein-related peptidase 4	19q13.41	4.25	8.52E-06	7
945	CD33	CD33 molecule	19q13.3	1.75	4.84E-05	7
5671	PSG3	pregnancy specific beta-1-glycoprotein 3	19q13.2	1.41	4.43E-03	7
**112398**	**EGLN2**	**egl nine homolog 2 (C. elegans)**	**19q13.2**	**1.16**	**6.49E-03**	**7**
11012	KLK11	kallikrein-related peptidase 11	19q13.3-q13.4	1.38	9.53E-03	7
5673	PSG5	pregnancy specific beta-1-glycoprotein 5	19q13.2	2.05	3.26E-06	6
22933	SIRT2	sirtuin (silent mating type information regulation 2	19q13	1.34	1.39E-05	6
1087	CEACAM7	carcinoembryonic antigen-related cell adhesion m	19q13.2	1.23	1.35E-03	6
1548	CYP2A6	cytochrome P450, family 2, subfamily A, polypepti	19q13.2	1.2	1.84E-03	6
1088	CEACAM8	carcinoembryonic antigen-related cell adhesion m	19q13.2	1.12	5.69E-03	6
5670	PSG2	pregnancy specific beta-1-glycoprotein 2	19q13.1-q13.2	2.35	1.63E-06	5
3817	KLK2	kallikrein-related peptidase 2	19q13.41	1.18	2.80E-04	5
282618	IL29	interleukin 29 (interferon, lambda 1)	19q13.13	1.5	5.82E-04	5
5582	PRKCG	protein kinase C, gamma	19q13.4	1.18	2.09E-03	5
1553	CYP2A13	cytochrome P450, family 2, subfamily A, polypepti	19q13.2	1.81	1.69E-06	4
57787	MARK4	MAP/microtubule affinity-regulating kinase 4	19q13.3	1.36	6.45E-06	4
26085	KLK13	kallikrein-related peptidase 13	19q13.3-q13.4	1.55	2.02E-05	4
282616	IL28A	interleukin 28A (interferon, lambda 2)	19q13.13	1.53	8.77E-05	4
2358	FPRL1	formyl peptide receptor-like 1	19q13.3-q13.4	1.13	8.06E-03	4
59348	ZNF350	zinc finger protein 350	19q13.33	1.5	2.26E-06	3
29998	GLTSCR1	glioma tumor suppressor candidate region gene 1	19q13.3	1.3	4.84E-06	3
10859	LILRB1	leukocyte immunoglobulin-like receptor, subfamily	19q13.4	1.38	5.89E-04	3
933	CD22	CD22 molecule	19q13.1	1.24	7.41E-04	3
25818	KLK5	kallikrein-related peptidase 5	19q13.3-q13.4	1.32	9.77E-04	3
9437	NCR1	natural cytotoxicity triggering receptor 1	19q13.42	1.25	1.99E-03	3
6795	AURKC	aurora kinase C	19q13.43	1.27	2.71E-03	3
27036	SIGLEC7	sialic acid binding Ig-like lectin 7	19q13.3	1.23	2.89E-03	3
5680	PSG11	pregnancy specific beta-1-glycoprotein 11	19q13.2	2.32	1.78E-07	2
92609	TIMM50	translocase of inner mitochondrial membrane 50 h	19q13.2	1.58	1.56E-05	2
55554	KLK15	kallikrein-related peptidase 15	19q13.41	3.05	4.92E-05	2
79784	MYH14	myosin, heavy chain 14	19q13.33	1.4	2.10E-04	2
11184	MAP4K1	mitogen-activated protein kinase kinase kinase kir	19q13.1-q13.4	1.5	5.12E-04	2
10849	CD3EAP	CD3e molecule, epsilon associated protein	19q13.3	1.21	2.07E-03	2
3802	KIR2DL1	killer cell immunoglobulin-like receptor, two domai	19q13.4	1.28	2.46E-03	2
284366	KLK9	kallikrein-related peptidase 9	19q13.33	1.27	2.74E-03	2
11006	LILRB4	leukocyte immunoglobulin-like receptor, subfamily	19q13.4	1.41	4.18E-03	2
11284	PNKP	polynucleotide kinase 3'-phosphatase	19q13.3-q13.4	1.27	4.68E-03	2
10298	PAK4	p21(CDKN1A)-activated kinase 4	19q13.2	1.19	6.50E-03	2

55786	ZNF415	zinc finger protein 415	19q13.41	2.5	1.33E-11	1
84911	ZNF382	zinc finger protein 382	19q13.12	1.69	8.49E-08	1
1340	COX6B1	cytochrome c oxidase subunit Vib polypeptide 1 (u	19q13.1	1.42	8.04E-07	1
4696	NDUFA3	NADH dehydrogenase (ubiquinone) 1 alpha subco	19q13.42	1.71	8.67E-07	1
84798	C19orf48	chromosome 19 open reading frame 48	19q13.33	1.63	2.61E-06	1
374907	B3GNT8	UDP-GlcNAc:betaGal beta-1,3-N-acetylglucosami	19q13.2	1.64	9.64E-06	1
162972	ZNF550	zinc finger protein 550	19q13.43	1.34	1.67E-05	1
56971	CEACAM19	carcinoembryonic antigen-related cell adhesion m	19q13.31	1.33	4.11E-05	1
201516	ZSCAN4	zinc finger and SCAN domain containing 4	19q13.43	1.37	6.31E-05	1
126272	EID2B	EP300 interacting inhibitor of differentiation 2B	19q13.2	1.51	7.73E-05	1
24139	EML2	echinoderm microtubule associated protein like 2	19q13.32	1.78	1.26E-04	1
11025	LILRB3	leukocyte immunoglobulin-like receptor, subfamily	19q13.4	1.56	1.66E-04	1
162966	ZNF600	zinc finger protein 600	19q13.41	1.31	2.48E-04	1
58506	SCAF1	SR-related CTD-associated factor 1	19q13.3-q13.4	1.48	3.25E-04	1
3809	KIR2DS4	killer cell immunoglobulin-like receptor, two domai	19q13.4	1.33	3.69E-04	1
51276	ZNF571	zinc finger protein 571	19q13.12	1.47	6.15E-04	1
3748	KCNC3	potassium voltage-gated channel, Shaw-related su	19q13.3-q13.4	1.36	7.22E-04	1
3783	KCNN4	potassium intermediate/small conductance calciur	19q13.2	1.35	7.35E-04	1
27335	EIF3K	eukaryotic translation initiation factor 3, subunit K	19q13.2	1.36	7.55E-04	1
112724	RDH13	retinol dehydrogenase 13 (all-trans/9-cis)	19q13.42	1.29	8.25E-04	1
94059	LENG9	leukocyte receptor cluster (LRC) member 9	19q13.4	1.36	1.01E-03	1
1549	CYP2A7	cytochrome P450, family 2, subfamily A, polypepti	19q13.2	1.65	1.04E-03	1
51545	ZNF581	zinc finger protein 581	19q13.42	1.26	1.27E-03	1
3904	LAIR2	leukocyte-associated immunoglobulin-like recepto	19q13.4	1.18	1.36E-03	1
6822	SULT2A1	sulfotransferase family, cytosolic, 2A, dehydroepia	19q13.3	1.11	1.42E-03	1
27202	GPR77	G protein-coupled receptor 77	19q13.33	1.28	1.99E-03	1
147945	NLRP4	NLR family, pyrin domain containing 4	19q13.42	1.34	2.22E-03	1
6183	MRPS12	mitochondrial ribosomal protein S12	19q13.1-q13.2	1.31	2.40E-03	1
4909	NTF5	neurotrophin 5 (neurotrophin 4/5)	19q13.3	1.26	2.73E-03	1
3816	KLK1	kallikrein 1	19q13.3	3.4	2.90E-03	1
3812	KIR3DL2	killer cell immunoglobulin-like receptor, three dom	19q13.4	1.3	3.06E-03	1
333	APLP1	amyloid beta (A4) precursor-like protein 1	19q13.1	1.32	3.12E-03	1
2906	GRIN2D	glutamate receptor, ionotropic, N-methyl D-aspart	19q13.1-qter	1.2	3.12E-03	1
60385	TSKS	testis-specific kinase substrate	19q13.3	1.23	4.28E-03	1
58491	ZNF71	zinc finger protein 71	19q13.4	1.17	6.75E-03	1
29844	TFPT	TCF3 (E2A) fusion partner (in childhood Leukemia	19q13	1.21	8.38E-03	1
199713	NLRP7	NLR family, pyrin domain containing 7	19q13.42	1.17	9.45E-03	1
84787	SUV420H2	suppressor of variegation 4-20 homolog 2 (Drosop	19q13.42	1.32	9.68E-03	1
3804	KIR2DL3	killer cell immunoglobulin-like receptor, two domai	19q13.4	1.77	1.15E-08	0
28968	SLC6A16	solute carrier family 6, member 16	19q13.1-q13.4	2.97	1.81E-07	0
587	BCAT2	branched chain aminotransferase 2, mitochondria	19q13	1.72	2.44E-07	0
284358	FLJ36070	likely ortholog of MEF2-activating SAP transcriptic	19q13.33	2.03	1.99E-06	0
147657	ZNF480	zinc finger protein 480	19q13.33	1.37	2.13E-06	0
284309	ZNF776	zinc finger protein 776	19q13.43	1.51	4.35E-06	0
55659	ZNF416	zinc finger protein 416	19q13.4	1.34	4.46E-06	0
90317	ZNF616	zinc finger protein 616	19q13.33	1.31	4.52E-06	0
147808	ZNF784	zinc finger protein 784	19q13.42	1.52	4.77E-06	0
57126	CD177	CD177 molecule	19q13.2	1.63	5.81E-06	0
284402	LOC284402	hypothetical protein LOC284402	19q13.11	1.38	7.02E-06	0
22847	ZNF507	zinc finger protein 507	19q13.11	1.48	8.07E-06	0
79891	ZNF671	zinc finger protein 671	19q13.43	1.52	9.54E-06	0
25888	ZNF473	zinc finger protein 473	19q13.33	1.34	1.40E-05	0
79760	GEMIN7	gem (nuclear organelle) associated protein 7	19q13.32	1.26	1.80E-05	0
342926	ZNF677	zinc finger protein 677	19q13.41	1.28	1.81E-05	0
342945	ZSCAN22	zinc finger and SCAN domain containing 22	19q13.43	1.28	2.36E-05	0
163115	ZNF781	zinc finger protein 781	19q13.12	1.38	2.42E-05	0
56344	CABP5	calcium binding protein 5	19q13.33	1.37	4.07E-05	0
147948	ZNF582	zinc finger protein 582	19q13.43	1.37	4.46E-05	0
1158	CKM	creatine kinase, muscle	19q13.2-q13.3	1.69	4.84E-05	0
148109	TMEM162	transmembrane protein 162	19q13.12	1.3	5.30E-05	0
115703	SNX26	sorting nexin 26	19q13.12	1.41	5.66E-05	0
147920	IGFL2	IGF-like family member 2	19q13.32	1.36	6.62E-05	0
81492	RSHL1	radial spokehead-like 1	19q13.3	1.39	7.28E-05	0
57663	USP29	ubiquitin specific peptidase 29	19q13.43	1.5	7.67E-05	0
85569	GALP	galanin-like peptide precursor	19q13.42	1.61	7.87E-05	0
7733	ZNF180	zinc finger protein 180	19q13.2	1.35	8.26E-05	0
27300	ZNF544	zinc finger protein 544	19q13.43	1.6	8.44E-05	0
11133	KPTN	kaptin (actin binding protein)	19q13.32	1.26	9.02E-05	0
6324	SCN1B	sodium channel, voltage-gated, type I, beta	19q13.1	1.55	9.06E-05	0
126433	FBXO27	F-box protein 27	19q13.2	1.35	9.06E-05	0
147719	LYPD4	LY6/PLAUR domain containing 4	19q13.2	1.36	9.22E-05	0
57030	SLC17A7	solute carrier family 17 (sodium-dependent inorga	19q13	1.33	1.02E-04	0

6543	SLC8A2	solute carrier family 8 (sodium-calcium exchanger	19q13.3	1.28	1.37E-04	0
147872	FLJ32658	hypothetical protein FLJ32658	19q13.33	1.67	1.37E-04	0
126299	ZNF428	zinc finger protein 428	19q13.31	1.44	1.54E-04	0
339344	LOC339344	hypothetical protein LOC339344	19q13.32	1.4	1.61E-04	0
54938	SARS2	seryl-tRNA synthetase 2, mitochondrial	19q13.2	1.5	1.66E-04	0
284346	ZNF575	zinc finger protein 575	19q13.31	1.32	1.80E-04	0
126248	WDR88	WD repeat domain 88	19q13.11	1.14	1.86E-04	0
946	SIGLEC6	sialic acid binding Ig-like lectin 6	19q13.3	1.32	1.90E-04	0
7710	ZNF154	zinc finger protein 154	19q13.4	1.19	2.24E-04	0
163071	ZNF114	zinc finger protein 114	19q13.32	1.4	2.39E-04	0
7728	ZNF175	zinc finger protein 175	19q13.4	1.44	2.51E-04	0
27033	ZBTB32	zinc finger and BTB domain containing 32	19q13.1	1.2	3.17E-04	0
84922	FIZ1	FLT3-interacting zinc finger 1	19q13.42	1.28	3.19E-04	0
8193	DPF1	D4, zinc and double PHD fingers family 1	19q13.13-q13.2	1.42	3.21E-04	0
284307	ZIK1	zinc finger protein interacting with K protein 1 hom	19q13.43	1.22	3.22E-04	0
9040	UBE2M	ubiquitin-conjugating enzyme E2M (UBC12 homol	19q13.43	1.47	4.18E-04	0
27120	DKKL1	dickkopf-like 1 (soggy)	19q13.33	1.44	4.62E-04	0
199720	GGN	gametogenetin	19q13.2	1.36	4.76E-04	0
317701	VN1R2	vomeronasal 1 receptor 2	19q13.41	1.23	5.11E-04	0
7638	ZNF221	zinc finger protein 221	19q13.2	2.34	5.17E-04	0
79744	ZNF419	zinc finger protein 419	19q13.43	1.38	5.47E-04	0
140612	ZFP28	zinc finger protein 28 homolog (mouse)	NA	1.37	5.72E-04	0
80110	ZNF614	zinc finger protein 614	19q13.33	1.19	6.11E-04	0
10520	ZNF211	zinc finger protein 211	19q13.4	1.43	6.62E-04	0
55957	LIN37	lin-37 homolog (C. elegans)	19q13.1	1.43	6.85E-04	0
84306	PDCD2L	programmed cell death 2-like	19q13.11	1.22	7.47E-04	0
57191	VN1R1	vomeronasal 1 receptor 1	19q13.4	1.26	7.70E-04	0
59283	CACNG8	calcium channel, voltage-dependent, gamma subu	19q13.4	1.77	7.95E-04	0
353355	ZNF233	zinc finger protein 233	19q13.31	1.47	8.33E-04	0
147699	FLJ40125	hypothetical protein FLJ40125	19q13.32	1.32	8.85E-04	0
147746	HIPK4	homeodomain interacting protein kinase 4	19q13.2	1.13	1.01E-03	0
374900	ZNF568	zinc finger protein 568	19q13.12	1.36	1.05E-03	0
59285	CACNG6	calcium channel, voltage-dependent, gamma subu	19q13.4	1.2	1.06E-03	0
79414	LRFN3	leucine rich repeat and fibronectin type III domain	19q13.12	1.29	1.07E-03	0
374918	IGFL1	IGF-like family member 1	19q13.32	1.27	1.09E-03	0
147923	ZNF420	zinc finger protein 420	19q13.12	1.3	1.11E-03	0
163255	ZNF540	zinc finger protein 540	19q13.12	1.27	1.11E-03	0
23619	ZIM2	zinc finger, imprinted 2	19q13.4	1.27	1.14E-03	0
1406	CRX	cone-rod homeobox	19q13.3	1.19	1.16E-03	0
7137	TNNI3	troponin I type 3 (cardiac)	19q13.4	1.17	1.24E-03	0
84964	ALKBH6	alkB, alkylation repair homolog 6 (E. coli)	19q13.12	1.39	1.30E-03	0
64763	ZNF574	zinc finger protein 574	19q13.2	1.4	1.53E-03	0
25850	ZNF345	zinc finger protein 345	19q13.12	1.46	1.76E-03	0
10794	ZNF460	zinc finger protein 460	19q13.4	1.14	1.83E-03	0
56917	MEIS3	Meis homeobox 3	19q13.32	1.47	1.84E-03	0
284323	ZNF780A	zinc finger protein 780A	19q13.2	1.19	1.86E-03	0
84446	BRSK1	BR serine/threonine kinase 1	19q13.4	1.26	1.90E-03	0
199745	THAP8	THAP domain containing 8	19q13.12	1.21	1.91E-03	0
84775	ZNF607	zinc finger protein 607	19q13.1	1.26	1.92E-03	0
162979	ZNF342	zinc finger protein 342	19q13.32	1.4	1.96E-03	0
7673	ZNF222	zinc finger protein 222	19q13.2	1.3	1.97E-03	0
23354	KIAA0841	KIAA0841	19q13.12	1.18	2.11E-03	0
57711	ZNF529	zinc finger protein 529	19q13.13	1.49	2.11E-03	0
148266	ZNF569	zinc finger protein 569	19q13.12	1.22	2.22E-03	0
64100	ELSPBP1	epididymal sperm binding protein 1	19q13.32	1.19	2.26E-03	0
57828	C19orf15	chromosome 19 open reading frame 15	19q13.1	1.22	2.31E-03	0
7771	ZNF228	zinc finger protein 228	19q13.2	1.32	2.60E-03	0
79147	FKRP	fukutin related protein	19q13.32	1.2	2.62E-03	0
388569	ZNF324B	zinc finger protein 324B	19q13.43	1.24	2.74E-03	0
148014	TTC9B	tetratricopeptide repeat domain 9B	19q13.2	1.37	2.86E-03	0
7288	TULP2	tubby like protein 2	19q13.1	1.22	2.95E-03	0
284370	ZNF615	zinc finger protein 615	19q13.33	1.28	3.11E-03	0
341	APOC1	apolipoprotein C-I	19q13.2	1.92	3.21E-03	0
54814	QPCTL	glutaminyl-peptide cyclotransferase-like	19q13.32	1.18	3.24E-03	0
26291	FGF21	fibroblast growth factor 21	19q13.1-qter	1.24	3.30E-03	0
199746	U2AF1L4	U2 small nuclear RNA auxiliary factor 1-like 4	19q13.12	1.27	3.51E-03	0
147912	SIX5	SIX homeobox 5	19q13.32	1.36	3.89E-03	0
80207	OPA3	optic atrophy 3 (autosomal recessive, with chorea	19q13.32	1.41	4.59E-03	0
92283	ZNF461	zinc finger protein 461	19q13.12	1.09	4.92E-03	0
126123	C19orf41	chromosome 19 open reading frame 41	19q13.33	1.33	5.49E-03	0
56301	SLC7A10	solute carrier family 7, (neutral amino acid transpo	19q13.1	1.27	5.58E-03	0
342897	LOC342897	similar to F-box only protein 2	19q13.2	1.54	5.74E-03	0

593	BCKDHA	branched chain keto acid dehydrogenase E1, alpl	19q13.1-q13.2	1.51	6.11E-03	0
8541	PPFIA3	protein tyrosine phosphatase, receptor type, f poly	19q13.33	1.17	6.46E-03	0
171169	SPACA4	sperm acrosome associated 4	19q13.33	1.32	6.86E-03	0
126526	C19orf47	chromosome 19 open reading frame 47	19q13.2	1.17	7.47E-03	0
163175	LGI4	leucine-rich repeat LGI family, member 4	NA	1.23	7.68E-03	0
199704	ZNF585A	zinc finger protein 585A	19q13.12	1.37	7.76E-03	0
495	ATP4A	ATPase, H+/K+ exchanging, alpha polypeptide	19q13.1	1.21	8.63E-03	0
342898	SYCN	syncollin	19q13.2	1.19	9.03E-03	0
126205	NLRP8	NLR family, pyrin domain containing 8	19q13.42	1.14	9.81E-03	0
55095	SAMD4B	sterile alpha motif domain containing 4B	19q13.2	1.19	9.89E-03	0
7691	ZNF132	zinc finger protein 132	19q13.4	1.27	9.98E-03	0
6633	SNRPD2	small nuclear ribonucleoprotein D2 polypeptide 16	19q13.2	1.24	9.99E-03	0

[a]Genes that mapped to chromosome 19q were examined for differential expression between renal oncocytoma and non-diseased kidney as as described in the Materials and Methods. Shown are adjusted significance values determined using the false discovery rate (FDR) method
[b,c]For each gene, the PubMed database was searched to identify publications that referenced the gene. For each publication, the Medical Subject Headings (MeSH) terms were searched for references to [c]cancer or [d]specifically to renal/kidney cancer.
